# DNACPR Decisions: Aligning Law, Guidance, and Practice

**DOI:** 10.1093/medlaw/fwac007

**Published:** 2022-04-29

**Authors:** Sabine Michalowski, Wayne Martin

**Affiliations:** School of Law, University of Essex, Colchester CO4 3SQ, UK; School of Philosophy and Art History, University of Essex, Colchester CO4 3SQ, UK

**Keywords:** Consultation, COVID-19, DNACPR forms, DNACPR decisions, Futility, ReSPECT framework

## Abstract

Do not attempt cardiopulmonary resuscitation (DNACPR) decisions are a means to consider in advance the appropriateness of CPR measures if an acute crisis arises. During the COVID-19 pandemic, problems with such decisions, for example the putting in place of DNACPR decisions for all residents of certain care homes, received a lot of attention, prompting a Care Quality Commission (CQC) report with recommendations for improvement. Building on the CQC report, our article addresses a cluster of legal uncertainties surrounding DNACPR decisions, in particular about the grounds for such decisions and the correct procedures for the legally required consultation, including with whom to consult.

This article will also analyse commonly used DNACPR forms, as well as the Recommended Summary Plan for Emergency Care and Treatment (ReSPECT) form, which aims to incorporate DNACPR decisions as part of more holistic end-of-life care planning. The analysis shows that all forms exhibit shortcomings in reflecting the legal requirements for DNACPR decisions. We recommend a number of changes to the forms aimed at rendering DNACPR practice compliant with the law and more protective of the person’s human rights.

## I. INTRODUCTION

In the 1960s, it became possible to apply cardiopulmonary resuscitation (CPR) when a person stops breathing or her heart stops beating. While CPR can save lives, it has a relatively low success rate (ranging from 15% to 20% when carried out in a hospital setting and from 5% to 10% if administered elsewhere) and can cause injuries such as punctured lungs, broken ribs, and bruising. Its administration therefore requires a risk–benefit assessment and CPR might not be indicated if there is no realistic chance that it will be successful in reviving the patient, if the risks outweigh the benefits, or if the person does not want it.^1^ Do not attempt cardiopulmonary resuscitation (DNACPR) decisions are a means to consider in advance the appropriateness of CPR measures for individual persons. As decisions about CPR, by their nature, need to be made in acute crisis situations and under enormous time pressure, the purpose of DNACPR decisions is to record in advance whether or not CPR should be initiated, and to avoid resort to CPR in circumstances where it would be inappropriate to administer it.

DNACPR decisions have always raised practical, ethical, and legal problems.^2^ As evidenced by the cases of *Tracey*^3^ and *Winspear*,^4^ one of the most prominent legal issues at the centre of litigation is that of consultation: to what extent do DNACPR decisions need to be made in consultation with the person concerned and/or an appropriate other, which we understand to include a welfare attorney, court ordered deputy, a relative or friend, or an Independent Mental Capacity Advocate. DNACPR decisions also give rise to ethical controversy because they can be seen as entailing the judgement that someone’s life is not worth saving. Legally, this translates into difficult questions about the criteria that inform DNACPR decisions, particularly where quality of life is a factor.^5^ There have also been repeated concerns that DNACPR decisions might be poorly understood by healthcare professionals. For example, several studies have shown that some interpret DNACPR decisions in a person’s medical records to indicate that life-sustaining measures other than CPR should not be attempted.^6^

A 2017 *BMJ* article summarized the predicament of DNACPR decisions as follows:

Clinicians are presented with an ethical dilemma: if they do not discuss CPR with a patient and record a decision, the patient may receive CPR that doesn’t work or that results in a quality of life that may not be acceptable to them; if they do, others may misinterpret it and compromise the patient’s overall care.^7^

As early as in 2014, following a review of DNACPR decisions that highlighted a range of problems, a meeting of patients, clinicians, healthcare commissioners, and regulators concluded that ‘patient and family involvement in decisions needed improving and that resuscitation decisions should be considered in the context of overall treatment plans.’^8^ As a consequence, the Recommended Summary Plan for Emergency Care and Treatment (ReSPECT) was introduced, which moves from DNACPR decisions made in isolation to integrating them into a more holistic personalised end-of-life care plan. ReSPECT makes recommendations for a person’s clinical care and provides the space to explore their choices regarding treatment in a future emergency in which they might not be able to make or express them.^9^ No uniform practice exists in this regard and while some care homes and hospitals apply the ReSPECT framework, others use DNACPR forms to record decisions that are limited to the administration of CPR.

The COVID pandemic brought renewed interest in DNACPR decisions and demonstrated that endemic problems might intensify in times of health crises. Especially during the early stages of the pandemic, DNACPR decisions received a lot of media attention, prompting an investigation and an extensive report from the Care Quality Commission (CQC).^10^ The CQC report identified widespread improper practice in the adoption of DNACPR decisions. Of particular concern to the CQC were reports of the so-called blanket use of DNACPR forms without individualised assessments, for example, for all residents of a particular care home, or groups of persons with shared characteristics, such as age or disability, consequent concerns about discrimination, and renewed concerns about failure of consultation.^11^

Our own data collected through a survey of frontline professionals and focus groups (the parameters of which are reported elsewhere^12^) reinforce the concerns identified by the CQC regarding the ways in which DNACPR forms are introduced into patient records. In addition, they show continuing confusion among frontline professionals as to the meaning and scope of DNACPR decisions and their appropriate use, both during and prior to the pandemic.^13^

It seems, then, that DNACPR decisions raise many problems, despite clarification in case law of the need for consultation, the development of the ReSPECT framework, longstanding and continuing academic and professional discussion, and updated and extensive professional guidelines on DNACPR issued jointly by the British Medical Association, the Resuscitation Council (UK) and the Royal College of Nursing (hereinafter: Guidance on CPR).^14^ The CQC report made recommendations for improvement. In line with its endorsement of the ReSPECT framework, it recommended that ‘DNACPR decisions need to be recognised as part of wider conversations about advance care planning and end of life care.’^15^ Another recommendation was that ‘[p]eople, their families and/or representatives, clinicians, professionals and workers need to be supported so that they all share the same understanding and expectations for DNACPR decisions.’^16^ Moreover:

[t]here must be comprehensive records of conversations with, and decisions agreed with, people, their families and/or representatives that support them to move around the system well. This requires providers to ensure standards of documentation and record keeping and sharing of information around the system.^17^

The recommendations made in the CQC report are important and welcome. But if reform of DNACPR practices is to be effective, clarity on the relevant legal standards is needed. Unfortunately, as this article will show, there continues to be uncertainty around key concepts that are fundamental for DNACPR decisions. Current guidance and case law indicate, for example, that futility is one ground for a DNACPR decision. But how should the concept of futility be understood and applied in this context? Moreover, forms used for recording DNACPR decisions in the National Health Service) NHS are naturally read as instructions and are often referred to as ‘DNACPR orders’. Are they really binding decisions? And what is their actual legal status? Thirdly, the courts have made clear that consultation is required in making DNACPR decisions. But what should such consultation consist in, and who should be involved? Does this depend on whether a DNACPR decision is strictly an exercise of clinical judgement or implicates broader issues about quality of life?

In what follows, this article will closely analyse these and related issues. It will also show that not only the DNACPR forms that the ReSPECT form is aiming to replace, but also the ReSPECT form itself have some shortcomings in reflecting the legal requirements on DNACPR decisions. In light of this, even though the CQC report rightly emphasises the centrality of documentation, we will argue that it is not sufficient to focus attention on record-keeping and documentation, without making sure that the forms on which these decisions are recorded are adequate and invite good and lawful practice. Using the ReSPECT form^18^ and two sets of DNACPR forms that are currently used in the NHS^19^ (the lilac^20^ and the red^21^ forms) as the basis for our analysis, we make recommendations on how the forms would need to be changed in order to achieve this.

## II. THE LEGAL NATURE OF DNACPR DECISIONS

In case of a cardiac or pulmonary arrest, a decision on whether or not to administer CPR needs to be taken instantly, leaving no time for lengthy best interests considerations or the possibility for conversations with the person involved or with their next of kin or carers. This is where DNACPR decisions come in. Their purpose is to assess, prior to cardiac or respiratory arrest occurring, whether CPR should be attempted or the patient left to die. The Guidance on CPR suggests initiating conversations about CPR if cardiorespiratory arrest is predicted or reasonably foreseeable, in order to consider in advance how to deal with such a future emergency. This can take place either as a conversation that is limited to the specific scenario of a future need for CPR or in the context of wider advance care planning.^22^

For example, the ReSPECT process recommended by the Resuscitation Council UK^23^ and endorsed by the CQC report^24^ is aimed at developing personalised recommendations for a person’s clinical care, based on an exploration of their views regarding end-of-life care, including what treatment they may or may not want in a future emergency in which they might not be able to make or express choices. The process results in an agreed care plan recorded on a ReSPECT form, of which recommendations on the administration of CPR are one element. According to the Resuscitation Council, the ReSPECT framework is increasingly adopted in care contexts,^25^ but different care homes and hospitals approach this question differently and some use DNACPR forms that are limited to the administration of CPR instead.

So how should the decisions recorded on these forms be made? Treatment decisions always need to be personalised and take into account all the circumstances of the individual case. The practice of issuing blanket DNACPR decisions for all residents of a care home or for a group of persons with shared characteristic, such as age or disability, without carrying out individualised assessments of each person’s situation, is thus clearly unlawful.^26^

One oddity of DNACPR recommendations is that they have at their core a decision *not* to provide treatment. Only decisions to provide treatment require a defence against a potential battery action—either in the form of consent, where the individual concerned has capacity,^27^ or in the form of the best interests tests, where this is not the case (section 5 of the Mental Capacity Act (MCA)). This means that, in principle, the person’s consent to a DNACPR decision is not required, and that the best interests test laid down in section 4 of the MCA 2005 does not apply to the decision not to administer CPR. As the Supreme Court explained in *Aintree*:

the focus is on whether it is in the patient’s best interests to give the treatment, rather than on whether it is in his best interests to withhold or withdraw it. If the treatment is not in his best interests … it will follow that it will be lawful to withhold or withdraw it. Indeed, it will follow that it will not be lawful to give it. It also follows that (provided of course that they have acted reasonably and without negligence) the clinical team will not be in breach of any duty towards the patient if they withhold or withdraw it.^28^

While neither the consent requirement nor the best interests test of the MCA, therefore, apply to DNACPR decisions, they do apply to the decision to administer CPR. A decision to withhold or withdraw CPR presupposes that the option to provide it has been assessed, resulting in the opinion that it is not indicated in the best interests of the person and that the health professional is therefore under no duty to provide it.^29^

When making such decisions, it must be borne in mind that English and Welsh law applies a strong presumption in favour of saving life.^30^ Accordingly, in case of cardiac or pulmonary arrest, there will be a presumption in favour of attempting CPR.^31^ In expressing a judgement that administering CPR would not be in the best interests of the person, DNACPR decisions apply narrowly and specifically to the withholding of CPR.^32^ To use them as a basis for denying other life-saving measures constitutes an unlawful departure from their authorised use. The lilac form makes this clear when it clarifies that ‘all other appropriate treatment and care will be provided’.

Another important question is whether a DNACPR decision binds the health professional who is faced with a person in cardiopulmonary arrest. The lilac forms states categorically that ‘in the event of cardiac or respiratory arrest, no attempts at CPR will be made’, while the red form equally categorically says in bold letters at its top ‘Do not attempt to resuscitate’. While none of the forms expressly refer to DNACPR decisions as orders, this is how many texts, including the leading medical law textbooks^33^ and articles in medical journals^34^ refer to these decisions. The Guidance on CPR at some point calls advance DNACPR decisions instructions.^35^ Both terms—order and instruction—could suggest that DNACPR forms record binding decisions. If that was the case, there would not only be no need, but not even room for a contemporaneous assessment at the scene of whether CPR should be administered in case of a cardiopulmonary arrest. They would rather be bound to follow the form.

However, from a legal perspective, the only situation in which an advance decision not to administer CPR is binding is where someone refuses to consent to CPR in an advance directive.^36^ To administer CPR in such a case would amount to a battery.^37^ Here, it becomes important to understand the differences between DNACPR decisions and advance CPR refusals. The law on binding advance treatment refusals can be found in sections 24–26 of the MCA 2005. An advance refusal is a decision made by the person whose treatment is governed by it and requires capacity (section 24(1) of the MCA). Specific form requirements apply to advance directives that refer to life-sustaining medical treatment, as would be the case with CPR. In such a scenario, section 25(5)(a) of the MCA requires that ‘the decision is verified by a statement by P to the effect that it is to apply to that treatment even if life is at risk’ and the form requirements of section 25(6) of the MCA are complied with:

- it is in writing;
- it is signed by P or by another person in P's presence and by P's direction;
- the signature is made or acknowledged by P in the presence of a witness; and
- the witness signs it, or acknowledges his signature, in P's presence.

Neither the DNACPR forms are currently in use in England and Wales, nor the ReSPECT form comply with these form requirements, and they can therefore not be regarded as binding treatment refusals, even where they record the person’s wishes not to receive CPR.

Strictly speaking, DNACPR forms record the assessment of a person’s best interests at a certain point in time that CPR should not be administered at a later point, but they are not binding and rather provide guidance to the person who needs to make a decision on CPR in an emergency situation. The ReSPECT form rightly refers to a DNACPR *recommendation* and makes clear right at the beginning that the form is not a legally binding document. The Guidance on CPR also emphasises this, even though at some point referring to DNACPR decisions as ‘instructions’. To call them orders or instructions is misleading and risks fostering misunderstanding of their legal status.

This raises the questions of what nomenclature is more appropriate. We refer to DNACPR *decisions*, but it is important to be clear about what decision is being referred to. In essence, a DNACPR form communicates the judgement of one health professional,^38^ the signatory, to another, the attending care-provider at the time of possible future cardiopulmonary arrest. The person attending in the crisis situation should take the signatory’s recommendation into account when making their own clinical judgement at the moment of the arrest. However, this does not exempt them from having to form their own clinical judgement on how to proceed.^39^ Indeed, best interests decisions on medical treatment need to assess the best interests of the person at the moment the treatment is needed.^40^ For example, the attending care provider should take into account whether cardiac arrest has occurred as part of a foreseen dying process or because of an unforeseen event that could be reversed through successful CPR and that might not have been contemplated when the form was signed. It also needs to be assessed whether the patient’s prognosis changed since the DNACPR decision was made.^41^

As can be seen, advance treatment refusals and DNACPR decisions are governed by different legal principles. Where both are in place, it is important to consider the relationship between the two. The DNACPR forms adopt different approaches in this regard. The ReSPECT form includes at its top a question about the existence and location of other relevant care planning documents, including advance refusals to consent to CPR. Further down on the form, where DNACPR decisions are recorded, no reference to the potential existence of a binding CPR refusal is made, suggesting that the two decisions are regarded as independent of one another. The red and lilac forms, on the other hand, list a valid advance CPR refusal as one of the reasons for which a DNACPR decision can be recorded, the lilac form requesting that a copy of that decision be attached to the DNACPR form.

From a legal perspective, it is not clear why advance refusals would be a reason to make a DNACPR decision. DNACPR decisions are non-binding decisions made by health professionals, in certain circumstances jointly with the person concerned. They play a role where CPR is regarded either as lacking a prospect of success or as overly burdensome, as will be discussed in more detail below. The forms are only signed by a health professional. Advance CPR refusals, on the other hand, are decisions solely of the person concerned and, as an expression of their autonomy, can be based on whatever consideration that person regards as relevant for refusing CPR. They can be broad, refusing CPR in all circumstances, or narrow, refusing it only in particular situations, e.g. in the context of a particular illness, or at a specific point in time, e.g. in the context of imminent medical treatment. Even though there might be good reasons to make a DNACPR decision even where an advance CPR refusal is in place and vice versa, given the different focus and remit of these two decisions, they should not be conflated.

It might be regarded as good practice to note on a DNACPR form the existence of an advance CPR refusal, as all three forms do. However, mere note of the existence of such a form is only helpful to the health professional having to deal with someone’s cardiopulmonary arrest if the advance refusal is either attached, as the lilac form requests, or if the DNACPR form notes the circumstances in which the advance refusal is aimed to apply. This way, the attending health professional can see from a glance at the DNACPR form whether a binding CPR refusal applies and/or a DNACPR decision is in place. From a legal point of view, a DNACPR decision only becomes relevant if no advance CPR refusal exists.^42^

## III. REASONS FOR MAKING DNACPR DECISIONS

That all DNACPR forms are only signed by a health professional, not jointly by the health professional and the individual or their appropriate other, might suggest that the decision recorded is at least primarily a decision of the health professional, made for clinical reasons. It is not clear, then, what role, if any, the wishes and values of the person concerned play in the decision-making process. The person concerned has a right to be consulted and the clinical decision needs to be made ‘in the light of the likely outcome, the patient’s ascertainable wishes, and his or her human rights’.^43^ Some academics go as far as to suggest that ‘the wishes of the person who wants to be resuscitated irrespective of the prognosis should be respected^’^,^44^ which might seem to require a health professional to administer CPR against their clinical judgement if the patient so wishes. On the other hand, the courts^45^ and clinical guidance^46^ insist that a person cannot require health professionals to administer treatment they do not regard as clinically indicated. So how can respect for the person’s wishes and for the professional’s clinical judgement be reconciled in case of disagreement?

The Guidance on CPR distinguishes two different scenarios in this respect. It suggests that ‘[i]f CPR may be successful in re-starting a person’s heart and breathing for a sustained period’,^47^ the benefits and burdens of CPR need to be balanced, which is not solely a clinical decision and should rather, where the person has capacity, be made jointly between the person and the health professionals, as the individual’s views on their overall circumstances and what burdens they are willing to assume are important.^48^ Indeed, the Guidance on CPR goes as far as to suggest that when ‘a person has decided that the quality of life that can reasonably be expected is acceptable to them, their wish for CPR should be respected’ and where clinicians feel unable to do so, a second opinion should be sought.^49^ On the other hand, where CPR does not have a realistic prospect of success, no risk/benefit assessment is necessary and the decision is presented by the Guidance as purely clinical.^50^

A distinction between DNACPR decisions based on a lack of a realistic prospect of success and those made on the basis of broader best interests considerations thus has important implications.

### A. CPR does not have a Realistic Prospect of Success

In case law, discussions around the lack of success of life-sustaining measures are frequently held under the notion of the concept of futility. In what follows we therefore refer both to *lack of a prospect of success* (the language used in the Guidance on CPR and on the DNACPR forms themselves) and to *futility* (as the concept that dominates the legal discussion of the issue).

The application of the concept of futility to end-of-life decision-making is complex and controversial. Indeed, futility lacks a clear definition, though Lord Goff’s treatment of futility in *Bland* has importantly shaped the legal use of the concept. The question in *Bland* was whether continuing life-sustaining artificial nutrition and hydration were indicated in the best interests of Anthony Bland, who was in a persistent vegetative state (PVS). Lord Goff stated that:

I cannot see that medical treatment is appropriate or requisite simply to prolong a patient's life, when such treatment has no therapeutic purpose of any kind, as where it is futile because the patient is unconscious and there is no prospect of any improvement in his condition. … [I]n the end, in a case such as the present, it is the futility of the treatment which justifies its termination. I do not consider that, in circumstances such as these, a doctor is required to initiate or to continue life-prolonging treatment or care in the best interests of his patient. It follows that no such duty [exists] in the case of Anthony Bland, whose condition is in reality no more than a living death, and for whom such treatment or care would, in medical terms, be futile.^51^

Thus, futility was not assessed with regard to whether the treatment at issue, in this case artificial nutrition and hydration, had a chance of success in terms of achieving its immediate and physiological purpose, i.e. to keep Anthony Bland alive, which it clearly did. Rather, his continued existence in PVS was regarded as futile, given that he was unconscious and there was no chance of improvement.^52^ Indeed, according to *Bland*, once a clinical diagnosis of PVS is made, all life-prolonging treatment is regarded as futile:

there is in reality no weighing operation to be performed. Here the condition of the patient … is such that life-prolonging treatment is properly regarded as being, in medical terms, useless … [I]t is futile because the patient is unconscious and there is no prospect of any improvement in his condition.^53^

Given that the narrow clinical purpose of the treatment at issue could have been achieved, the way the concept of futility is applied clearly implies a quality-of-life decision.

The Supreme Court also discussed the concept of futility in *Aintree*. In the Court of Appeal, Sir Alan Ward suggested that an analysis of futility should go beyond an assessment of the effectiveness of the treatment in question for ‘coping with the current crisis’ and also evaluate ‘the improvement, or lack of improvement which the treatment would bring to the general health of the patient’.^54^ In the Supreme Court, Lady Hale instead expressed the view that the trial judge ‘was correct to consider whether the proposed treatments would be futile in the sense of being either ineffective or of no benefit to the patient’.^55^ She went on to say that:

it is setting the goal too high to say that treatment is futile unless it has “a real prospect of curing or at least palliating the life-threatening disease or illness from which the patient is suffering”. … A treatment may bring some benefit to the patient even though it has no effect upon the underlying disease or disability.^56^

Lady Hale also stressed that ‘the assessment of the medical effects of the treatment [was] only part of the equation. Regard had to be had to the patient's welfare in the widest sense’.^57^ Thus, this definition of futility considers not only whether treatment has a clinical chance of success, but also whether it might bring benefits, including non-medical benefits, to the patient.

Other courts applied futility with a narrow focus on the immediate function of the treatment in question, and thus its clinical effectiveness, which is only one of two parts of the futility definition applied in *Bland* and *Aintree*. For example, a tracheostomy was not regarded as futile because it could achieve its purpose of substituting for a compromised cough reflex and clear secretions.^58^ Equally, renal replacement therapy was not regarded as futile, since it kept the patient alive.^59^ These decisions make a clear distinction between clinical futility and treatment that might not be indicated for other reasons. There is thus not a single and uniformly accepted and applied legal definition of futility. Indeed, the very fact that the concept was interpreted differently by all three courts involved in the *Aintree* decision highlights its conceptual uncertainties.^60^

The Guidance on CPR does not explicitly refer to the concept of futility and rather focuses on whether CPR has no realistic prospect of success. Whether or not this is the case is regarded as a purely clinical decision that does not require any best interests or quality-of-life considerations. Just like with futility, a judgement as to whether there is a prospect of success depends on how success is defined and measured, i.e. whether the yardstick is the lack of effectiveness of the treatment or the lack of benefits for the patient. The second prong of futility as defined in *Aintree*, the lack of any benefit to the person, leaves room for including the person concerned in the decision-making process, given Lady Hale’s emphasis on benefits including other than medical benefits.^61^ This broader definition of futility therefore does not give rise to a situation where only clinical considerations guide the treatment decision. Rather, only futility where treatment is ineffective coincides with the scenario in which the Guidance on CPR regards a decision as purely clinical and obviates the need for any balancing of interests or quality-of-life considerations.

However, even this narrow understanding of futility or lack of prospect of success leaves room for interpretation. Clinical judgement almost always involves probabilities instead of certainties. This is inherent in the way the Guidance on CPR refers to the situation in which CPR requires only a clinical judgement: lack of a realistic prospect of success. The qualifier ‘realistic’ shows that lack of success does not need to be a certain outcome in order to justify that the decision on DNACPR is regarded as purely clinical. However, as soon as such uncertainty comes in, it is no longer clear why a decision as to whether or not to attempt CPR should be exclusively in the domain of clinical knowledge. As long as there is *some* chance of success, even if only remote, the potential arises for disagreement as to whether that chance is worth taking, even at the risk of causing serious harm or distress in the process. For example, some persons, whether for religious or other reasons, may be quite clear in wishing for any and all treatment options to be exhausted, no matter how invasive, provided that there is some prospect that the treatment will be successful. While this does not mean that health professionals are therefore bound to offer that treatment if they regard it as futile, it must be carefully considered under what circumstances a prediction of a lack of a prospect of success of CPR can be made. It also needs to be considered whether the wishes of the person or their appropriate others should be disregarded where there is at least a slight chance that CPR might be successful.

Given these uncertainties, while it is important to consider the effectiveness and prospect of CPR to achieve its immediate clinical purpose as part of an assessment of whether it is appropriate to administer it, it is questionable that a health professional’s assessment that there is no realistic prospect of success excludes the need to take into account the views and interests of the person.^62^

None of the DNACPR forms expressly uses the language of futility. On the lilac form, one of the reasons for which a DNACR form can be signed is that CPR is *unlikely to be successful*, but no explanation is provided as to how likelihood is to be understood or evaluated. The red form mentions as a first reason why CPR would be inappropriate that there is *no realistic possibility of CPR succeeding*. It adds that in this scenario, the decision is a unilateral medical decision. While this coincides with the view promoted in the Guidance on CPR, it is problematic for the reasons explained above. Neither form provides an explanation for how success of the treatment should be assessed. Should the *Bland* and *Aintree* definition of futility be applied, measuring success according to whether or not it would be ‘ineffective or … of no benefit to the patient’? Or is the determining issue whether CPR has a chance of achieving its clinical purpose of resuscitating the person?

### B. DNACPR Decision Based on Balancing Risks and Benefits

According to the Guidance on CPR, outside of cases with a lack of a realistic chance of success, CPR is not indicated where the balancing of all the risks and benefits involved comes down against administering it. As this would include an assessment of the overall quality of life to which the person would be restored, the Guidance acknowledges that the decision thus goes beyond merely clinical considerations and should therefore be made jointly with the person, at least if she has capacity.^63^

The MCA Code of Practice distinguishes further between cases where CPR is overly burdensome and where there is a lack of prospect of recovery.^64^ Case law often discusses these concepts together. As seen above, Lady Hale, for example, stated that

it is setting the goal too high to say that treatment is futile unless it has ‘a real prospect of curing or at least palliating the life-threatening disease or illness from which the patient is suffering’. … A treatment may bring some benefit to the patient even though it has no effect upon the underlying disease or disability.^65^

Thus, where a person is afflicted with a disability or an incurable illness, a DNACPR decision cannot be justified simply on the basis that resuscitation would restore the person to the disabled or ill state they were in before suffering a cardiopulmonary arrest, but not to a state of good health without disability. Also, CPR cannot be withheld purely because the person does not have a chance of recovering fully from the underlying illness that might have caused the cardiac arrest. Instead, in all these scenarios, the appropriateness of CPR depends on an overall assessment of the quality of life the person would likely experience after a successful administration of CPR.

The lilac form lists as one of the reasons for a DNACPR decision that CPR may be successful but followed by a length and quality of life which would not be of overall benefit to the patient. The red form makes no reference to benefits and quality of life, but it provides as one of three possible grounds for a DNACPR decision that the patient’s condition indicates that CPR would have an uncertain outcome and that, after discussion, it was agreed that CPR would not be appropriate. It is not clear on what basis an uncertain outcome would be determined and whether this decision would have any link with quality-of-life considerations. Moreover, the appeal to the uncertainty of outcome is both confusing and worrying, given that the outcome of CPR is always uncertain.

None of the forms make any reference to joint decision-making. Thus, none of them reflect the suggestion, made by the Guidance on CPR and cited with approval by the Court of Appeal in *Tracey*, that if ‘a person has decided that the quality of life that can reasonably be expected is acceptable to them, their wish for CPR should be respected.’^66^ But even if the forms did include this, as would be desirable, this would lead to the difficult question of what would happen where the clinical opinion and that of the person concerned diverge, given that health professionals cannot be required to administer treatment against their clinical judgement. There are several possibilities for dealing with this issue. The solution proposed by the Guidance on CPR is to arrange for a second opinion.^67^ But what if that does not resolve the disagreement? It seems that the clinician providing the second opinion has the last word: they can decide not to make the DNACPR decision or, if they agree with the first opinion, no third decision needs to be sought, so the original decision would stand. While reluctant to offer a definitive view on whether or not there is a right to a second opinion, either under the common law or Article 8(1) of the ECHR, the Court of Appeal accepted the submission in *Tracey* that

there is no obligation to offer to arrange a second opinion in a case, such as that of Mrs Tracey, where the patient is being advised and treated by a multi-disciplinary team all of whom take the view that a DNACPR notice is appropriate.^68^

That in the end, clinical opinion seems to prevail in case of disagreement contradicts the Guidance on CPR’s position that the person’s decision should be followed even if there is only a small chance of success. The main reason for this seems to be that a clinician cannot be obliged to administer CPR against her clinical judgement. However, bearing in mind that those who put in place the DNACPR decision will often not be the same professionals who need to apply it in case of an arrest, the justification for putting it in place against the person’s wishes could then not be an exercise of their right not to administer treatment against their clinical judgement. Whether the principle goes as far as making a strong recommendation to others not to administer CPR because to do so would go against the clinical judgement of the health professional signing the DNACPR decision is at least questionable. Indeed, it is not clear in what circumstances it would be suitable to put in place such a decision against the wishes of the person with capacity. The appropriateness of such a decision should therefore be carefully considered and recorded.

However, where a DNACPR decision is made under these conditions, it seems important to record on the DNACPR form that there was a disagreement and what the different views were, so that the health professional who needs to decide whether to administer CPR in case of an arrest is privy to this information. It should also be noted whether the person is seeking another medical opinion on the issue and, if so, that the DNACPR decision needs to be reviewed in light of that.

The Guidance on CPR limits the cases of joint decision-making to persons with capacity. Stressing the need to avoid quality-of-life assessments that reflect the views of the health professionals or the next of kin or carers, it emphasises that the relevant consideration needs to be ‘whether the patient (if they had capacity) would regard the level of possible recovery acceptable, taking into account the invasiveness of CPR and its likelihood of success’.^69^ This reflects general medical law principles that if the person lacks capacity to make their own treatment decision, the decision needs to be made in their best interests, giving due weight to their wishes and beliefs,^70^ which is part of the healthcare professional’s duty of care towards that person. This includes seeking the person’s own views where possible as well as those of appropriate others on the person’s quality of life and best interests, a point we return to below in the context of a discussion of the consultation requirement.

## IV. CONSULTATION

In this section, we will first explain the legal requirements on consultation, followed by an analysis of whether and how the forms reflect them.

### A. The Right to be Consulted

If healthcare professionals intend to put a DNACPR decision in place, the affected individuals, and, in case of their inability to make healthcare decisions and express their views, an appropriate other, need to be informed and consulted about the decision.^71^ In *Tracey*, the Court of Appeal made clear that an individual’s right to private life under Article 8(1) of the ECHR is engaged when DNACPR decisions are being made, as a

decision as to how to pass the closing days and moments of one’s life and how one manages one’s death touches in the most immediate and obvious way a patient's personal autonomy, integrity, dignity and quality of life.^72^

Consultation is also a duty under common law.^73^ As a DNACPR decision is a life and death decision, the individual ‘is entitled to know that such an important clinical decision has been taken’.^74^ As Ryder LJ explained in *Tracey*,

[t]he duty to consult is integral to the procedural obligation to ensure effective respect for the article 8 right, without which the safeguard may become illusory and the interest may not be reflected in the clinical judgment being exercised.^75^

This applies whether or not CPR is regarded as futile:

The fact that the clinician considers that CPR will not work means that the patient cannot require him to provide it. It does not, however, mean that the patient is not entitled to know that the clinical decision has been taken.^76^

Indeed, individuals can only be involved in their own care and, for example, reach the conclusion to refuse CPR, if they are told about DNACPR decisions that are being considered and informed of the reasons for them.^77^ Also, not to be told about the decision would deprive the individual of the opportunity to seek a second opinion.^78^

In *Tracey*, Lord Dyson emphasised that

since a DNACPR decision is one which will potentially deprive the patient of life-saving treatment, there is a presumption in favour of patient involvement. There need to be convincing reasons not to involve the patient.^79^

This would, in particular, be the case where consultation ‘is likely to cause her to suffer physical or psychological harm’,^80^ as opposed to mere distress.^81^ While the distinction between psychological harm and distress might not always be easy, the onus is thus on those who do not involve the person or their appropriate other to rebut the presumption in favour of consultation. In a different context, that of reflecting on the extent of the duty to disclose risks of a procedure in order to avoid a negligence action, the Supreme Court equally highlighted that withholding information from a person that is necessary to make an informed decision is only lawful if the health professional ‘reasonably considers that its disclosure would be seriously detrimental to the patient's health’.^82^ However, the Supreme Court stressed the exceptional nature of relying on this ‘therapeutic exception’ and emphasised that

it is not intended to subvert that principle by enabling the doctor to prevent the patient from making an informed choice where she is liable to make a choice which the doctor considers to be contrary to her best interests.^83^

*Winspear* made clear that the Article 8(1) of the ECHR and common law duties to consult are also owed to persons who lack capacity. According to the court, section 4(7) of the MCA then becomes relevant for the question of who should be consulted. This means that the views of anyone named by the person to be consulted, engaged in caring for the person or interested in their welfare, donee of a lasting power of attorney or court appointed deputies must be taken into account, where consulting them is practicable and appropriate.^84^ Where consultation that would be appropriate and practicable is omitted, ‘the decision to file the DNACPR notice on the patient’s medical records would be procedurally flawed’^85^ and therefore not in accordance with the law, as required by Article 8(2) in order to justify the resulting interference with Article 8(1) of the ECHR.

*Winspear* concerned a man who was so severely disabled from birth that he never had capacity and it was impossible to communicate with him about his wishes, feelings, values, and beliefs. This explains the decision’s exclusive focus on consultations with others, in this case the mother, according to section 4(7) of the MCA. It is worrying that this seems to have been interpreted to mean more generally that where someone lacks capacity, consultation must only take place with those mentioned in section 4(7) of the MCA. For example, the Guidance on CPR dedicates one section to communication and discussion with patients with capacity, followed by a section entitled ‘communication and discussion with those close to a patient who lacks capacity’.^86^ It is automatically assumed that lack of capacity means that communication with the persons themselves is not necessary. However, this violates the spirit and the letter of the MCA that aims to empower persons who lack capacity to be involved in decisions, including medical decisions. This is reflected in section 4(4) of the MCA, which requires,

so far as reasonably practicable, [to] permit and encourage the person to participate, or to improve his ability to participate, as fully as possible in any act done for him and any decision affecting him.

It is crucial not to lose sight of this imperative and not to turn exclusively to appropriate others where someone lacks capacity, instead of attempting to involve them in the discussions.

In addition to the questions about whom to consult, it needs to be asked what is meant by consultation. In *Tracey*, the Court of Appeal referred to a presumption in favour of involvement, which seems to indicate more than simply informing the persons or their relatives of a decision unilaterally made by the physician. According to Ryder LJ, it ‘involves a discussion, where practicable, about the patient's wishes and feelings.’^87^ However, it is not entirely clear what type of involvement and discussion are required.^88^

The courts did not set different standards for consultation depending on the reasons for which a DNACPR decision is made. The Guidance on CPR, on the other hand, applies its distinction between DNACPR decisions that are made because there is no realistic prospect of success and those where CPR would be overly burdensome. In the former case, while it is recommended that time be allowed for discussion and reflection, the focus of consultation is on informing the person or an appropriate other of the decision and the reasons behind it.^89^ This is probably because such decisions are regarded as merely clinical and not requiring any weighing or balancing of the wishes of the person.

If CPR is regarded as overly burdensome, the Guidance on CPR distinguishes between persons with or without capacity. Those who have capacity

should be offered information about CPR, about the local resuscitation policy and services, and about their role in decision-making in relation to CPR. In order to determine whether the benefits of CPR would be likely to outweigh the harms and burdens, or whether the level of recovery expected would be acceptable to the patient, there should be sensitive exploration of the patient’s wishes, feelings, beliefs and values.^90^

With regard to persons who lack capacity, the Guidance on CPR highlights the legal obligation to consult with those close to the patient, omitting any reference to the fact that it is necessary to involve the person herself as much as possible. Instead, it only refers to that person’s previously expressed wishes and values.^91^ Where a personal welfare attorney or a court-ordered deputy are in place, ‘[t]he aim should be, whenever practicable and appropriate, to explain, discuss and agree the intended plan of treatment for the patient, including whether or not to attempt CPR.’^92^ As already explained, this does not reflect the legal requirement that a person without capacity be included in decisions as much as reasonably practicable.

### B. Recording Consultation on the Forms

When considering to what extent the DNACPR forms reflect the law on consultation, it is worth noting that the lilac form summarises the Article 8 implications on its back, while the red form does not provide any information about the human rights relevance of consultation. The lilac form distinguishes between DNACPR decisions adopted because CPR is likely to be unsuccessful and those where CPR, if successful, would be followed by a length or quality of life that is not of benefit to the person. Where the DNACPR decision is based on a lack of prospect of success, it provides tick boxes to record whether or not the decision has been discussed with the person or whether their relevant others were informed of it. The form also asks for reasons why this might not have been done. Thus, where CPR is unlikely to be successful, the form requires discussion with the person with capacity and the mere provision of information to appropriate others if he/she lacks capacity. No reasons are given for this distinction. Neither is there an explanation of what a discussion needs to entail beyond providing information. Equally, the forms do not clarify what could be acceptable reasons for not carrying out a consultation.

With regard to DNACPR decisions based on quality-of-life considerations, the lilac form asks whether the person was involved in the discussion. Where a welfare attorney has been appointed, her involvement is requested. Otherwise, the health professional needs to specify on the form with whom the decision, made for the overall benefit of the person, was discussed. This suggests that it needs to be discussed with someone and that the mere provision of information would not be sufficient in this scenario.

The red form has one box with the rubric *decision and reasons why CPR would be inappropriate* and then provides a separate box to record communication, without imposing different communication or consultation requirements based on the reasons for the DNACPR decision. It provides yes or no tick boxes to state whether the decision has been discussed with the person and below the no box, two more boxes can be ticked: decision not discussed because the patient lacks capacity or because this might result in physical or psychological harm. There is no encouragement to record the content of the discussion or to give further information explaining why there was a risk of physical or psychological harm and how great that risk was assessed to be. There is also a tick box for whether or not the decision was discussed with a relevant other person, asking for detail but providing very little space for presenting it. No explanation is given as to the circumstances in which a discussion with relevant others would be necessary.

Given that the ReSPECT framework starts from the assumption that end-of-life planning is a holistic process based on conversations between the person herself and health professionals, of which a CPR decision is only one part, the form specifically asks to record the shared understanding of the person’s health and current condition. As already explained, CPR decisions are then noted as part of the clinical recommendations. Concerning who to consult, the ReSPECT form follows the Guidance on CPR, in that it asks for participation in the plan only of a person who has the mental capacity to do so, even if it can only be exercised with support. However, if the person does not have capacity, their participation is no longer mentioned, as section 4(4) of the MCA 2005 requires, but the focus rather shifts to ascertaining their past and present views and to consultation of their legal proxies, family, or friends. No distinction is made based on the reasons for the CPR decision.

## V. RECOMMENDATIONS AND CONCLUSIONS

The COVID pandemic brought renewed attention to DNACPR decisions, particularly in care homes, but the problems with DNACPR decisions predate the pandemic and cannot be expected to disappear with it. In the foregoing, we undertook an examination of the law, guidance, and administrative paperwork associated with the practice of DNACPR decisions. Our analysis brought to light areas of continuing legal uncertainty regarding DNACPR decisions, but also significant areas of misalignment among current legal standards, authoritative guidance, and the proformas used to record such decisions. We have shown, in particular, that the current forms used for DNACPR decisions do not adequately reflect the legal and professional requirements on DNACPR decision-making and do not invite the decision-maker to take all important factors that should guide these decisions into account.

An important point to note is that the red and lilac forms do not accurately or adequately reflect the legal status of a DNACPR decision, which is neither an order nor a legally binding decision. The commonly used forms present the recommendation not to attempt CPR in such categorical terms that it might be understood as an order, which is indeed the way the decision is often referred to in the legal and medical literature. Terminology is important in this context. If DNACPR forms, signed by ‘the most senior clinician responsible for the person’s care as defined explicitly by local policy’,^93^ are called orders or instructions or state unequivocally that no attempts at CPR will be made, this does not invite anybody, especially a potentially more junior person like a paramedic or junior doctor, to carry out their own clinical assessment when the emergency situation arises. Yet, in the absence of a binding advance CPR refusal, those charged with making the decision at the time of the arrest would breach their duty of care if they followed a DNACPR decision without making their own clinical assessment of all relevant circumstances at the time the decision is made. To flag this and avoid confusion as to the legal force and status of DNACPR decisions, it is important that DNACPR forms clearly identify them as recommendations, as the ReSPECT form does.

At the same time, given the difference between DNACPR decisions and binding advance treatment refusals, the two should not be conflated and the existence of an advance refusal should not be regarded as a reason for a health professional to put a DNACPR decision in place, as both the red and lilac forms suggest. When it is considered to be useful that a DNACPR decision is put in place side by side with an advance refusal, for example to record clinical reasons for such a recommendation or discussions between health professionals and patients that result in a joint view, the DNACPR form should record the existence of the advance refusal and its scope of application and that the two distinct records should be kept together.

Probably, the most difficult issues around DNACPR decisions concern the extent to which they should be regarded as strictly clinical decisions, and the role the persons concerned should have in these existential decisions about the end of their lives. We argued that when the basis for a DNACPR decision is the lack of a realistic prospect of success, the standard of success must be understood narrowly. In this context, success of CPR should be understood to mean success in achieving the physiological purpose of CPR: reviving the person—and not as a reference to the benefits. The latter decision requires quality-of-life considerations that make it necessary to take into account the person’s views and values and therefore transcends purely clinical considerations.

However, it was also shown that it will only be a very small number of cases in which a lack of success can be predicted with certainty, rather than based on a calculation of probabilities. Only in those exceptional cases can it be said that the decision is based purely on medical expertise which patients and their appropriate others will be lacking.

This is not to negate that there might be many situations in which the administration of CPR might be disproportionate to the potential gains, particularly given the low success rate and the high invasiveness of CPR, the inherent risks to the person’s physical integrity and the prolongation of an inevitable dying process in some cases, which might raise questions of whether CPR could then violate the clinician’s ethical duty of non-maleficence.^94^ Rather, this analysis aims to show that the moment probabilities come into the equation, a balancing of the likelihood of success or failure with other important factors, such as the potential burdens of CPR and the quality of the life that would potentially be restored, needs to be carried out.^95^ This requires involvement of the person and/or appropriate others. It should therefore be clarified in the Guidance on CPR that a DNACPR decision can only be regarded as purely clinical in exceptional cases where success of CPR can be excluded with certainty.

The forms should reflect this and not only ask to record that CPR would have no chance of success, but also explain that this means a certainty, rather than a probability, that CPR would not succeed in reanimating the person. They should also include a brief explanation as to why there is no chance of success. Even though these cases are regarded as purely clinical, consultation to determine the person’s wishes would still be required. This consultation should take place with the person herself if she has capacity. A person who lacks capacity should nevertheless be consulted wherever possible, as should relevant others in that case. Both the consultation and the person’s wishes should be recorded, as the ReSPECT form already requires.

In all other cases, the form should explain the reasons for which CPR is regarded by the clinician signing the form as overly burdensome or without prospect of recovery. However, the forms also need to draw attention to the view expressed by the Guidance on CPR that, at least where the person has capacity, the DNACPR decision is a joint decision between that person and the health professional. Where the person does not have capacity, it needs to be explored and recorded on the form what the person’s own views and/or those of appropriate others on this are. If the person or their appropriate others disagree with the clinical assessment on best interests, this should be recorded on the form, as well as whether a second opinion is being awaited. However, it should be carefully considered in all cases whether a DNACPR decision is appropriate in such circumstances, given the strong legal presumption in favour of saving life. The conflict that needs to be resolved in cases of disagreement is that between the person or their appropriate others’ assessment of the person’s best interests and quality of life with the health professionals’ freedom not to have to administer treatment against their clinical judgement. The latter scenario will, however, not often arise, as the DNACPR decision is frequently a recommendation to a different health professional.

Consideration should also be given to a further change as regards standards of consultation. As we have seen, current guidance establishes different standards for consultation, depending on the basis for the DNACPR decision. A lower level of consultation is required in cases where there is no reasonable prospect of success; more substantial consultation is expected where further elements implicating quality of life are a relevant consideration. But if lack of success can only rarely be predicted with certainty, we propose that similar consultation rules should apply to all DNACPR decisions, regardless of the grounds upon which they are made. Such an approach would not only simplify the process and its documentation, but also better reflect the importance a decision on withholding CPR has on the person.

To take seriously the purpose of the consultation requirement, that is, to involve the person or their next of kin in a particularly crucial treatment decision, consultation should provide the persons to be consulted with the relevant information and considerations on which the clinical judgement is based, explain the possible treatment options in order to enable meaningful involvement, or the lack of options in cases of futility, and provide space for exploration and discussion of the person’s wishes and values. This should be made more prominent on the forms, by requesting at least a brief summary of the consultation, what was discussed and what the views of the consulted persons were. It is also important to make clear that lack of capacity does not mean that the person concerned therefore does not need to be consulted. Rather, according to section 4(4) of the MCA, everything that is reasonably practicable needs to be done to ensure the person’s involvement in the decision-making process. To honour the importance of consultation, the DNACPR forms should make clear that consultation is a legal duty and a human right that exists regardless of whether CPR is futile or the person lacks capacity.

Could the problems with DNACPR decisions be overcome by a move from isolated DNACPR decision-making to adopting the more holistic and person-centred ReSPECT end-of-life care planning framework,^96^ as the CQC report seems to suggest? While a shift to the ReSPECT framework might significantly improve end-of-life care, it would still leave some problems unaddressed. Indeed, our analysis has shown that the ReSPECT form in quite a few regards does not reflect important legal standards and requirements, for example concerning the need to include the person who lacks capacity in the decision-making process and to note the reasons for which CPR is not recommended.

But are the forms used really so important that our insistence on their overhaul can be justified? We suggest that the forms are essential. Where forms exist to assist professionals with making and recording important and complex decisions, they are likely to understand these forms as setting the standards they need to comply with and as adequately reflecting what is required of them. Bad forms are therefore likely to lead to bad practice. While professionals might potentially be excused if they fill in the forms inappropriately and it is later found that the forms did not satisfy legal standards, the person whose rights are affected by DNACPR decisions needs to be protected against life-and-death decisions that do not take their rights sufficiently into account.

This article’s focus on legal problems around DNACPR decisions is not meant to suggest that the necessary culture change can be achieved through the law alone. Instead, it has to be acknowledged that more needs to be done to enable and encourage frank conversations about end-of-life care, including whether CPR would be appropriate in case of cardio-pulmonary arrest. The CQC report therefore rightly emphasised that

**Clinicians, professionals and workers must have the knowledge, skills and confidence to speak with people about, and support them in, making DNACPR decisions.** To do this, there needs to be clear and consistent training, standards, guidance and tools for the current and future workforce. This needs to be in line with a national, unified approach to DNACPR decision making.^97^

Nonetheless, legal clarity has a vital role to play in informing the training, standards, guidance, and tools needed to foster the much-needed changes for which the CQC has called.

## VI. ETHICS 

Ethical approval for the survey and focus groups was obtained by the University of Essex Humanities Subcommittee.

## Footnotes

1 British Medical Association, Resuscitation Council (UK) and Royal College of Nursing, *Decisions**Relating to**Cardiopulmonary**Resuscitation* (3rd edn, BMA, 2016) 7.

2 See, eg, J Wilson, ‘To What Extent Should Patients Be Included in Decisions Regarding Their Resuscitation Status?’ (2008) 34 J Med Ethics 353; Z Fritz and JP Fuld, ‘Ethical Issues Surrounding Do not Attempt Resuscitation Orders: Decisions, Discussions and Deleterious Effects’ (2010) 36 J Med Ethics 593; Z Fritz and JP Fuld, ‘Development of the Universal Form of Treatment Options (UFTO) as an Alternative to Do Not Attempt Cardiopulmonary Resuscitation (DNACPR) Orders: A Cross-Disciplinary Approach’ (2015) 21 J Eval Clin Pract 109; R Hakim and others, ‘Factors Associated With Do-not-Resuscitate Orders: Patients’ Preferences, Prognoses, and Physicians’ Judgments’ (1996) 125 Ann Int Med 284.

3
*Regina (Tracey) v Cambridge University Hospitals NHS Foundation Trust and another (Equality and Human Rights Commission and* *Another* *Intervening)* [2014] EWCA Civ 822.

4
*Winspear v City Hospitals Sunderland NHS Foundation Trust* [2015] EWHC 3250 (QB).

5 See, eg, *Aintree University Hospitals NHS Foundation Trust v James* [2013] UKSC 67; *Re R (Adult: Medical Treatment)* [1996] 2 FLR 99; *County of Durham & Darlington NHS Foundation Trust v PP* [2014] EWCOP 9.

6 See MC Beach and RS Morrison, ‘The Effect of Do-not-Resuscitate Orders on Physician Decision-Making’ (2002) 50 J Am Geriatr Soc 2057; EA Henneman and others, ‘Effect of Do-not-Resuscitate Orders on the Nursing Care of Critically Ill Patients’ (1994) 3 Am J Crit Care 467; JL Chen and others, ‘Impact of Do-not-Resuscitation Orders on Quality of Care Performance Measures in Patients Hospitalized with Acute Heart Failure’ (2008) 156 Am Heart J 78; RI Cohen and others, ‘The Impact of Do-not-Resuscitate Order on Triage Decisions to a Medical Intensive Care Unit’ (2009) 24 J Crit Care 11.

7 Z Fritz and others, ‘Resuscitation Policy Should Focus on the Patient, not the Decision’ (2017) 356 BMJ j813.

8 ibid.

9 For further information, see <www.resus.org.uk/respect> (last accessed on 26 March 2022).

10 Care Quality Commission, ‘Protect, Respect, Connect—Decisions about Living and Dying Well during COVID-19’ (2021) <https://www.cqc.org.uk/sites/default/files/20210318_dnacpr_printer-version.pdf> (last accessed on 26 March 2022).

11 ibid 11–12.

12 Kuylen K and others, ‘COVID-19 and the Mental Capacity Act in Care Homes: Perspectives from Capacity Professionals’ *Health and Social Care in the Community* 2022;00:1–1. *https://onlinelibrary.wiley.com/doi/epdf/10.1111/hsc.13747*.

13 Fitton E and others, Managing DNACPR Decisions in Residential Care:  Towards Improved Training for Social Care and Capacity Professionals (manuscript under review).

14 British Medical Association, Resuscitation Council (UK) and Royal College of Nursing (n 1).

15 Care Quality Commission (n 10) 6.

16 ibid 43.

17 ibid 7.

18 A specimen copy of the ReSPECT for can be found at <https://aace.org.uk/wp-content/uploads/2020/09/ReSPECT-v3-1-formSPECIMENFINAL.pdf> (last accessed on 26 March 2022).

19 Note, though, that the forms chosen are only samples and that many different versions of the lilac and red forms are available and used, sometimes with enormous differences from one form to the other. See: <http://dnar.co.uk/sample-dnar-forms-nhs-staff/> (last accessed on 26 March 2022).

20 For a sample, see, <https://www.oxfordshireccg.nhs.uk/professional-resources/documents/clinical-guidelines/end-of-life/DNACPR-printed-form.pdf> (last accessed on 26 March 2022).

21 For a sample, see <https://www.northamptongeneral.nhs.uk/Communications/Coronavirus/How-to-complete-the-DNACPR-form.pdf> (last accessed on 26 March2022).

22 British Medical Association, Resuscitation Council (UK) and Royal College of Nursing (n 1) 4.

23 Resuscitation Council UK, ‘ReSPECT’ (Resuscitation Council UK), <https://www.resus.org.uk/respect> (last accessed on 26 March 2022).

24 Care Quality Commission (n 10).

25 Resuscitation Council UK, ‘ReSPECT’ (n 23).

26 Care Quality Commission (n 10) 11.

27
*Airedale NHS Trust v Bland* [1993] AC 789 (HL) 882 (Lord Browne-Wilkinson).

28
*Aintree University Hospitals NHS Foundation Trust v James* (n 5) [22] (Lady Hale).

29
*Burke, R (on the application of) v General Medical Council & Ors* [2005] EWCA Civ 1003.

30
*Re T (Adult: Refusal of Treatment*) [1993] Fam 95 (CA) 112 (Donaldson MR).

31 British Medical Association, Resuscitation Council (UK) and Royal College of Nursing (n 1) 17.

32 ibid 10.

33 See, eg, E Jackson, *Medical Law* (5th edn, OUP 2019) 939; G Laurie, S Harman and E Dove, *Mason McCall Smith’s Law and Medical Ethics* (11th edn, OUP 2019) para 19.32.

34 R Griffith, ‘Do Not Attempt Resuscitation Orders in Primary Care Settings’ (2018) 6 Br J Community Nurs 252, 304.

35 British Medical Association, Resuscitation Council (UK) and Royal College of Nursing (n 1) 15.

36 C Foster and T Hope, ‘What Sort of DNAR Order Is That’ (2012) 105 J R Soc Med 279.

37
*Airedale NHS Trust v Bland* (n 27).

38 ‘The overall clinical responsibility for decisions about CPR, including DNACPR decisions, rests with the most senior clinician responsible for the person’s care as defined explicitly by local policy. This could be a consultant, general practitioner (GP) or suitably experienced and competent nurse’, see British Medical Association, Resuscitation Council (UK) and Royal College of Nursing (n 1) 26.

39 ibid 17.

40 Foster and Hope (n 36) 280.

41 ibid 282.

42 See also ibid 279–282.

43 Laurie, Harman and Dove(n 33) para 19.33.

44 ibid.

45 See *Regina (Tracey) v Cambridge University Hospitals* (n 3); *Aintree University Hospitals NHS Foundation Trust v James* (n 5); *Re R (Adult: Medical Treatment*) (1996) 3 FCR 473.

46 British Medical Association, Resuscitation Council (UK) and Royal College of Nursing (n 1) 12.

47 ibid 13.

48 ibid 13–14.

49 ibid 15.

50 ibid 12.

51
*Airedale NHS Trust v Bland* [1993] AC 789, 869 (Lord Goff).

52 For critical discussion see, in particular: JM Finnis, ‘Bland: Crossing the Rubicon?’ (1993) 109 L Q Rev 329; J Keown, ‘Restoring Moral and Intellectual Shape to the Law after Bland’ (1997) 113 L Q Rev 481.

53
*Airedale NHS Trust v Bland* (n 51).

54
*Aintree University Hospitals NHS Foundation Trust v James* [2013] EWCA Civ 65 [38] (Ward LJ).

55
*Aintree University Hospitals NHS Foundation Trust v James* (n 5) [40] (Lady Hale).

56 ibid [43] (Lady Hale).

57 ibid [41] (Lady Hale). For a discussion, see C Auckland, ‘A Façade of Futility’ (2021) 137 L Q Rev 451, 462.

58
*Abertawe Bro Morgannwg University Local Health Board v RY (by his litigation friend the Official Solicitor)* [2017] EWCOP 2 [53] (Hayden J).

59
*St George's Healthcare NHS Trust v P (by his litigation friend, the Official Solicitor*), Q [2015] EWCOP 42 [35] (Newton J).

60 For an overview of different approaches taken by different courts, see Auckland (n 57) 451.

61
*Aintree University Hospitals NHS Foundation Trust v James* (n 5) [41] (Lady Hale). For a discussion, see Auckland (n 57).

62 Auckland (n 57) 451, 465.

63 British Medical Association, Resuscitation Council (UK) and Royal College of Nursing (n 1) 13.

64 Department for Constitutional Affairs, *Mental Capacity Act 2005. Code of Practice* (TSO 2007) para 5.31.

65
*Aintree University Hospitals NHS Foundation Trust v James* (n 5) [43] (Lady Hale).

66 ibid 15; *Regina (Tracey) v Cambridge University Hospitals* (n 3) [61] (Lord Dyson MR).

67 British Medical Association, Resuscitation Council (UK) and Royal College of Nursing (n 1) 26.

68
*Regina (Tracey) v Cambridge University Hospitals* (n 3) [65] (Lord Dyson MR).

69 British Medical Association, Resuscitation Council (UK) and Royal College of Nursing (n 1) 16.

70 Mental Capacity Act 2005 s 4(4).

71 Department of Health and Social Care, *NHS Constitution for England* (2012) <https://www.gov.uk/government/publications/the-nhs-constitution-for-england/the-nhs-constitution-for-england> Principle 4 of the NHS Constitution (last accessed on 26 March 2022).

72
*Regina (Tracey) v Cambridge University Hospitals* (n 3) [32] (Lord Dyson MR).

73 ibid [89] (Longmore LJ); [94] (Ryder LJ).

74 ibid [55] (Lord Dyson MR).

75 ibid [95] (Ryder, LJ).

76 Lord Dyson MR (n 72).

77 ibid [97] (Ryder LJ).

78 (Lord Dyson MR) (n 72).

79 ibid [53] (Lord Dyson MR).

80 ibid [54] (Lord Dyson MR).

81 For a critical discussion of the Tracey requirement to disclose and discuss DNACPR decisions with patients, see S Cahill, ‘Should Human Rights and Autonomy be the Primary Determinants for the Disclosure of a Decision to Withhold Futile Resuscitation?’ (2019) 25 New Bioeth 39. For academic literature welcoming the decision, see Z Fritz and others, ‘DNACPR Decisions: Challenging and Changing Practice in the Wake of the *Tracey* Judgment’ (2014) 14 Clin Med 571.

82
*Montgomery v Lanarkshire Health Board* [2015] UKSC 11 [88] (Lord Kerr and Lord Reed).

83 ibid [91] (Lord Kerr and Lord Reed).

84
*Winspear v City Hospitals Sunderland* (n 4) [46] (Blake J).

85 ibid [47].

86 British Medical Association, Resuscitation Council (UK) and Royal College of Nursing (n 1) 16.

87
*Regina (Tracey) v Cambridge University Hospitals* (n 3) [99] (Ryder LJ).

88 Health professionals’ uncertainty on how to interpret the legal requirement is documented in FMA MacCormick and others, ‘Resuscitation Decisions at the End of Life: Medical Views and the Juridification of Practice’ (2018) 44 J Med Ethics 376.

89 British Medical Association, Resuscitation Council (UK) and Royal College of Nursing (n 1) 12–13.

90 ibid 15.

91 ibid 16.

92 ibid 19.

93 British Medical Association, Resuscitation Council (UK) and Royal College of Nursing (n 1) 26.

94 P Druwé and others, , on behalf of the REAPPROPRIATE study group, ‘Cardiopulmonary Resuscitation in Adults Over 80: Outcome and the Perception of Appropriateness by Clinicians’ (2020) 68 J Am Geriatr Soc 39, 40.

95 For a discussion of how results of clinical studies on the success rate of CPR can inform patient’s informed choices, see I Oving and others, ‘Comorbidity and Survival in the Pre-Hospital and In-Hospital Phase after Out-of-Hospital Cardiac Arrest’ (2020) 153 Resuscitation 58.

96 For a discussion of the advantages of integrating DNACPR decisions in holistic end-of-life care planning, though not with specific reference to the ReSPECT framework, see, eg, CC Hall and others, ‘CPR Decision-Making Conversations in the UK: An Integrative Review’ (2019) 9 BMJ Support Palliat Care 1; C Mockford and others, ‘DNACPR Orders: A Systematic Review of the Barriers and Facilitators of Decision-Making and Implementation’ (2015) 88 Resuscitation 99.

97 Care Quality Commission (n 10) 42.

